# Synucleins As Biomarkers of Severity in Autism Spectrum Disorder

**DOI:** 10.7759/cureus.69356

**Published:** 2024-09-13

**Authors:** Lal D. V. Nair, Senthil Kumar Sivanesan, Devika S Kumar

**Affiliations:** 1 Pediatrics, Saveetha Medical College and Hospital, Saveetha Institute of Medical and Technical Sciences, Saveetha University, Chennai, IND; 2 Research and Development, Saveetha Medical College and Hospital, Saveetha Institute of Medical and Technical Sciences, Saveetha University, Chennai, IND

**Keywords:** alpha-synuclein, autism biomarker, autism spectrum disorder (asd), beta-synuclein, childhood autism rate scale, circulating biomarker, serum biomarker

## Abstract

Introduction

Autism spectrum disorder (ASD) is a lifelong disorder affecting children quite early in life, manifested as delays in communication and stereotypic behaviors. To date, it is diagnosed clinically using the Diagnostic and Statistical Manual-5 (DSM-V) criteria due to the lack of biomarkers that can specifically denote the disorder. The role of synucleins in this context has been considered due to the increasing evidence of neurodegeneration in many autistic children. Synucleins are a group of soluble neuronal proteins primarily expressed in the central nervous system. They are of three types: α-, β-, and ɣ-synuclein. α-synuclein is involved in vesicle trafficking and release of neurotransmitters. There is no uniformity in the scientific community regarding their levels of autism, with few studies showing increasing levels and others to the contrary. Hence, the present study was conceived to analyze the levels of α-synuclein and β-synuclein in autistic children and to correlate with the disease severity.

Objectives

The main objective of the study was to assess the levels of α- and β-synuclein in autistic children of 2-8 years of age and to identify the correlation between the severity of core symptoms of autism and α- and β-synuclein levels. It is intended to assess the possibility of using α- and β-synuclein/their ratio as a biomarker of the severity of autism.

Materials and methods

Plasma levels of α-synuclein and β-synuclein were measured in 160 ASD children and 40 healthy age and sex-matched children by ELISA. Their symptom severity was assessed with CARS-2 ST and the Indian Scale of Autism Assessment (ISAA). Values of α- and β-synuclein were analyzed for correlation with the severity rating of ASD. Cut-off values of α-synuclein and β-synuclein that discriminate the presence of autism and its severity were assessed using Jamovi 2.4.14 software.

Results

The results show that α-synuclein levels were significantly reduced (5.02 ± 0.586; range: 3.13-6.0 ng/ml) when compared with healthy controls (29.47 ± 18.62 ng/ml; range: 22.39- 36.56) with p < 0.001, and β-synuclein levels were elevated (1424 ng/ml ± 122; range: 1229-1616 ng/ml) when compared to control, though not significantly. Plasma levels of α-synuclein significantly correlate with disease severity with good diagnostic accuracy (86%), but β-synuclein levels did not correlate with severity. The fold changes of synucleins, especially the fold decrease in levels of α-synuclein, were discriminative for the diagnosis and severity with good sensitivity (93.6%), specificity (74.3%), positive predictive (92.6%), and negative predictive values (76.5%). The fold increase in β-synuclein did not have any significance in predicting the severity of autism.

Conclusion

The present study showed that α-synuclein and β-synuclein were associated with ASD and can be used to assess its severity. A fold decrease in α-synuclein was found to have good discriminating value in differentiating the severity of autism. It may be of use especially in mild and high-functioning autism, when clinically distinguishing them may be difficult.

## Introduction

Autism spectrum disorder (ASD) is a neurodevelopmental disorder identified by deficits in social communications and interactions and the presence of repetitive or restricted behaviors with psychological or physiological co-morbidities, all present in the early developmental period and causing significant impairment in social or any other areas of current functioning. Of late, considerable efforts have been made to develop biomarkers and assessment tools for early detection [1]. Some studies have raised the possibility of synuclein derangements in ASD with contradictory findings concerning their values [2-4]. Synucleins are a group of small proteins that are soluble, found primarily in the nervous tissues of vertebrates, and have a common α-helical lipid binding motif. So far, three synucleins have been investigated: α-, β-, and γ-synucleins [2]. The α and β have 127 to 140 amino acids, of which 55-62% are identical sequences with similar domain organization, coded by three synuclein (SNC) genes subtyped as A, B, and C in chromosomes 4q23 (SNCA), 5q35 (SNCB), and 10q21 (SNCC) [5]. The critical role of α-synuclein in synaptic functions includes vesicular stabilization, maintenance of the synaptic pool and its plasticity, regulating dopamine synthesis, and neurotransmitter release [4]. The aggregation of α-synuclein happens through three mechanisms: microglial activations, impaired efflux of α-synuclein from the brain into circulation induced by high Cyclooxygenases-2 (COX2) and Prostaglandin-E2/E-type Prostanoid2 (PGE2/EP2), and PGE2/EP2 receptor shedding from the brain into plasma. Such a microglial activation results in presynaptic glutamate excitotoxicity due to the release of glutamate vesicles in the synapses, causing an extracellular buildup of glutamate and leading to neuronal death. Further, the toxicity is aggravated as the shedding of PGE2/EP2 receptors results in the loss of the neuroprotection of PGE2/EP2 [6]. It has been found that α-synuclein participates in synaptic vesicular exocytosis and endocytosis by modulating the vesicle docking, priming, and fusion. Overexpression of α-synuclein increases the docked dense core vesicles [6]. A sudden rise in α-synuclein inhibits synaptic vesicle recycling during significant stimulation (20 Hz) at the stimulated synapses. Here, extra α-synuclein was found to produce loss of synaptic vesicles and an expansion of the plasma membrane, suggesting impairment of recycling of vesicles [7]. It has been suggested that alterations in the expression and solubility of α-synuclein cause α-synuclein aggregation in many neurodegenerative disorders. A recent study identified that the decrease in α-synuclein correlates positively with disease severity, whereas few others contradict it by proving that the levels were increased in ASD [3,6]. The non-homogeneity of the study population and many factors, including the influence of sex parameters, interfere with the results of these studies [8]. Further, β-synuclein inhibits α-synuclein aggregation, both in vivo and in vitro [9]. In a study with a mouse model of Parkinson's disease, it was found that the overexpression of β-synuclein affected the reduction of α-synuclein expression at the protein level, indicating a possible correlation in the expression levels between these two synuclein proteins [10]. α- and β-synucleins are abundantly present in the central nervous system (CNS), while γ-synuclein is distributed predominantly in the peripheral nervous system [11]. Most of the studies reported absolute values of synucleins compared with controls. This may not bring the actual picture of change, especially during unit conversions, which may be needed when comparing the results from different products. Analyzing the fold-change in their values may reduce such disparities in the results. Further, considering the reported protective role of β-synuclein in controlling α-synuclein aggregation, assessing it in terms of fold change and a possible relation between them may bring about some close correlation with disease severity. This may be used as objective evidence for the clinical diagnosis and the effect of interventions. The present study aimed to examine potential associations between α-synuclein and β-synuclein levels in ASD children by assessing the changes in plasma levels and comparing them with the severity of symptoms.

The main objectives of the study were to assess the levels of α- and β-synuclein in autistic children of 2-8 years of age and to identify the correlation between the severity of core symptoms of autism and α- and β-synuclein levels. The study also intended to assess the possibility of using α- and β-synucleins or their ratio as a biomarker of the severity of autism.

## Materials and methods

Recruitment of participants

Children who came to the general outpatient department suspected of having speech or communication issues were assessed in the developmental clinic before being recruited for the prospective study. The study extended nearly three years, from June 2020 to March 2024, excluding the one year (January 2021-December 2021) of the COVID-19 pandemic when the study was halted temporarily. Subsequently, when the study was restarted, children with symptoms or serological evidence of COVID-19 infection or its known complications were identified and excluded. Written informed consent was obtained from the parents of all the children before the study, following the most recent Declaration of Helsinki. The Institutional Ethics Committee of Saveetha Medical College and Hospital approved the study (approval no.: 003/06/2021/IEC.SMCH). They were recruited from the Saveetha Child Development Centre at Saveetha Medical College and Hospital, Chennai, India, and the three Vistara CDC groups of child development centers attached to it.

Study design

This analytical cross-sectional study was designed with children screened for autism using valid tools like the Modified Checklist for Autism in Toddlers (MCHAT) for toddlers or the Trivandrum Autism Behaviour Checklist (TABC) for older children. A developmental and behavioral pediatrician (DBP) did the initial assessments at the outpatient department. Data was collected using a standard proforma containing details of current health, developmental history, family history, treatment history, and other illnesses. *Sampling and Sample Size* Power analysis was done using G Power software version 3.1.9.7 with an alpha of 0.5 and size effect of 0.25. The required sample size was 165, with an actual calculated power of 0.9503 with the sample size for the correlation. Two hundred children between two and eight years old were recruited for the study by convenient sampling. They were age and sex-matched into two groups: normal children (n = 40; control) and ASD children (n = 160; cases). *Inclusion Criteria* All the children between two and eight years who screened positive for ASD were included in the study as cases; children with no significant illness attending the outpatient department for purposes like general check-ups or vaccination were included as a control group. *Exclusion Criteria* A detailed history of common comorbidities, such as epilepsy, with a comprehensive medical observation and neuroimaging, genetics, metabolism, chromosome, and other related examinations were done to exclude children with Rett syndrome, Fragile X syndrome, genetic metabolic disorders, and other neurological conditions such as epilepsy. All known deficiency states like calcium, magnesium, vitamin B6, and D deficiencies were excluded clinically and by assessing their blood levels. Children with any significant recent infections, including COVID-19, measles, mumps, chickenpox, etc., up to one year before sample collection were excluded from the study. *Procedure* ASD children were evaluated by a DBP and a clinical psychologist with childhood autism rating scale-2 standard test (CARS-2 ST), Indian Scale of Autism Assessment (ISAA), and autism diagnostic inventory- revised (ADI-R) and satisfied with Diagnostic and Statistical Manual-5 (DSM-5) criteria. The DBP, after completing the CARS/ISAA, by observing the child’s behavior and conducting a parental interview after ruling out other comorbidities or illnesses, confirmed the diagnosis along with the clinical psychologist using ADI-R/DSM-5 on the day of the first visit itself in a quiet examination room with no distracting objects during the evaluation. The total process took an average of 1.5 hours per child, and for those who were uncooperative, a second meeting was rescheduled after one week. Blood samples were collected to rule out any deficiencies as described above as well as to measure α- and β-synuclein levels. Blood for α- and β-synucleins was analyzed using ELISA commercial kits and compared with the scoring of ASD severity levels. *Measurements of α- and β-Synuclein* Five ml of non-hemolyzed, non-hyperlipidemic, and non-icteric lithium-heparinized plasma was used in this study. Multiple aliquots of all blood samples were centrifuged; the plasma isolated was stored at -80°C till ELISA loading was decided. Double-antibody sandwich ELISA method was used to determine the plasma concentrations of α-synuclein and β-synuclein. ELISA commercial kits were used according to the manufacturer’s instructions (Human α-Synuclein Assay Kit-IBL, Code Number 27740, and Human β-synuclein ELISA Kit-Five Photon Biochemicals, part number hSNCB-Biotin (96T)). Using the sample buffer supplied along with the kit, the plasma samples were prepared to a 10-fold dilution to obtain adequate concentrations to measure. To increase accuracy, all plasma samples were analyzed in two independent experiments, with each experiment also performed in duplicate.

Standard and samples were added to the appropriate microtiter plate wells with a biotin-conjugated antibody specific to either α-synuclein/ β-synuclein (The microtiter plate provided in this kit had been pre-coated with an antibody specific to either α-synuclein/ β-synuclein). Next, avidin conjugated to horseradish peroxidase (HRP) was added to each microplate well and incubated. After the TMB substrate solution was added, only those wells that contained either α-synuclein/β-synuclein, biotin-conjugated antibody, or enzyme-conjugated avidin exhibited a color change. The addition of sulfuric acid solution terminated the enzyme-substrate reaction, and the color change was measured spectrophotometrically at a wavelength of 450 nm ± 10 nm. The concentration of α-synuclein/β-synuclein in the samples was then determined by comparing the optical density of the samples to the standard curve.

Assessments

*Screening for Autism* MCHAT: MCHAT is a validated screening tool for autism for children in the toddler age group. A "yes" response indicates a typical response, and "no" indicates an atypical response in most questions except 2, 5, and 12, which are reverse scored. A total score of more than eight indicates "high risk." TABC: TABC was developed and validated by the Child Development Centre (CDC), Kerala, India, for screening children above two years. It has a sensitivity of 80%, specificity of 91.1%, PPV of 36.36%, and NPV of 98.61%, thereby making it a good tool for screening Indian children of this age group. A score of 36-43 is taken as mild-moderate, and ≥44 is taken as severely autistic [12]. *Diagnostic Evaluation of ASD* Following the administration of the screening tools, to confirm the diagnosis of ASD, all the suspected children were assessed by a clinical psychologist using ADI-R and further satisfied with DSM-5 criteria by a trained DBP. It provided categorical results for three domains of language/communication, reciprocal social interactions, and repetitive behaviors/interests. Behaviors are scored from zero-behavior does not present to three-severe behavioral problems. The total of all three domains gives the cut-off for the diagnosis of autism. The cut-off for social interaction is 10, communication and language are 8 for verbal and 7 for nonverbal, and restricted repetitive behaviors are 3. An autism diagnosis is indicated when scores in all three behavioral areas meet or exceed the specified minimum cut-offs [13]. *Measurement of ASD Severity* The severity of symptoms of each ASD child was assessed by the DBP using both the Indian Scale for Assessment of Autism (ISAA) and the Childhood Autism Rating Scale-2 Standard Test (CARS-2 ST). CARS-2 ST: It is an observational screening tool with 15 items used to differentiate children with ASD. The symptoms were assessed on a scale from 1 (normal) to 4 (severe) for the 15 items suggestive of autism. Total scores range from a lowest of 15 to a highest of 60, with scores below 30 indicating no autism; scores between 30 and 36.5 indicating mild to moderate autism; and scores from 37 to 60 indicating severe autism [14]. Since the study was designed to make a comparative judgment of the level of autism-related behaviors in a group of autistic children and values of synucleins, T-scores or their elevation were also considered instead of categorical values like mild-moderate or severe autism alone. T-score values that correspond to total raw scores were taken. Total raw scores of CARS-2 ST have a mean of 50T and a standard deviation of 10T. Thus, the CARS-2 ST individual with a T-score of 50T exhibits an average level of autism-related behaviors compared to individuals diagnosed with autism. Someone with a T-score of 65T may be said to have a significantly higher level of such behaviors than the average autism-diagnosed individual, while someone with a T-score of 38T may be said to have a significantly lower level of such behaviors. The SEM of CARS-2 ST was 2.7T. Thus, the values were interpreted as follows: >70 as extreme levels, 60-70 as very high level, 55-59 as high level, 45-54 as average level, 40-44 as low level, 25-39 as very low level, and <25 as minimal-to-no symptoms of autism compared to those with an autism diagnosis. ISAA: ISAA is a well-validated tool with 40 items falling under six domains: social relationship and reciprocity; emotional responsiveness; speech, language, and communication; behavior patterns; sensory aspects; and cognitive components. These are rated with a Likert scale based on history and interviewer observation from 1 to 5, with an increasing order of severity. A score of <70 indicates no autism, 70-106 as mild autism, 107-153 as moderate autism, and >153 as severe autism. The psychometric properties of this tool, currently used by the Government of India to certify disability in autism, are excellent. It has a sensitivity of 93.3%, a specificity of 97.4%, positive and negative likelihood ratios of 85.7 and 98.7, and positive and negative predictive values of 35.5 and 0.08, respectively [15]. Thus, the severity rating of each child was observed against behaviors exhibited by autistic children (using T-scores of CARS-2 ST) and also against normal children (using ISAA).

Statistical analysis

Demographic data were analyzed with descriptive statistical methods and mean values with standard deviations were derived. The distribution of the emerging data of different blood parameters and their grading was analyzed for normality using the Shapiro-Wilk test. All the variables were nonparametrically distributed. Hence the results were presented as median along with 25th and 75th quartile values. Medians were compared with the Kruskal-Wallis ANOVA test. Post-hoc analysis was done using Dwass-Steel-Critchlow-Fligner (DSCF) pair-wise comparison of different grades of ISAA and CARS-2 ST with different blood markers. Statistical analysis was performed with Jamovi software, version 2.4.14. Being a nonparametric distribution, the outliers of the blood parameter markers (α and β synuclein, their fold changes, and β/α ratio) when compared with CARS 2ST/ISAA grades have been identified using box plots by IQR values. T-scores or their severity levels were considered for analysis rather than categorical values of CARS like mild-moderate or severe autism, as it gives better comparative judgments regarding autistic behaviors in the group since the ASD children diagnosed with ADI-R were only considered. T-score values that correspond to total raw scores were taken for the study. Correlation analysis of the different markers with the CARS2 ST and ISAA grades was done with Spearman's correlation (rho), considering the non-parametric data distribution and the presence of a few outliers. ROC curves were prepared for the values of synucleins and their fold changes to assess the cutoff values using Jamovi.2.4.14.

## Results

Demographic data

The children in the 2-8 year age group were mostly sex-matched; hence, male:female was not calculated. The mean age of children who were enrolled in the study was 3.82 years (46.82 months with an SD of 8.36); the minimum age was reported as two years and two months (26 months), and the maximum age was five years and eight months (68 months). However, sex distribution showed that 84 (53%) were males and 76 (47%) were females. The socioeconomic factor analysis revealed the majority were in the middle-income group (n = 132) and most were up to high school educated (n = 118) (Table 1). The main parental concern was the absence of name-call response and poor eye contact (n = 64; 40%), followed by speech delay (n = 50; 31.3%), overactive (n = 18; 11.2%), poor name-call response and eye contact but verbal with speech delay (n = 20; 12.5%), and speech delay with reduced clarity (n = 8; 5%).

**Table 1:** Demographic characteristics of study groups ^*^World Bank statistics 2023 - GNI per capita income of family in $ According to the latest statistics (2023) of the Government of India, available at the National Statistical Office, a family income of INR 5,00,000-30,00,000/GNI per capita of INR 113,144. Mother's education was considered in this study as educational status is biased against females in many Indian households and the academic status of the mother has a direct bearing on the training of the special needs children also. Our study found no significant difference between the educational status of ASD and control group parents. Of late, since the gap between the middle-income group and middle class has widened, we have considered GNI per capita and education status separately.

Parameters	ASD group	Control group
Mean age (months)	45.44 ± 8.32 SD	46.32 ± 7.96 SD
Minimum age (months)	26	28
Maximum age (months)	68	65
Socioeconomic status		
Middle income^*^ ($1,146 to $14,005)	132	35
Low income^*^ (=$1,146)	28	5
Mother’s educational status		
High school	118	38
Middle school	42	12

The antenatal period was uneventful in 119 (74.4%), but 21 (13.1%) mothers each had gestational diabetes and pregnancy-induced hypertension, and 20 (12.5%) had other conditions. However, there was birth asphyxia with delayed cry requiring resuscitation and difficult/prolonged second stage of labor in 52 (32.5%). None were products of multiple pregnancies. There was no significant family history, with 78 (48.7%) ASD children falling first in birth order and 82 (51.3%) second in order of birth. The mean maternal age was 28 with an SD ±2.56 with a CI of 19-35 years.

Severity rating

The majority of children (n = 118; 73.8%) had severe autism, 26 (16.3%) had mild-to-moderate autism, and 16 (10%) had mild-to-no symptoms of autism, according to the CARS-2 ST raw score-based severity rating. The median ISAA score was 148 (IQR: 95-201) with 70 (43.75%) reported as moderate, 55 (34.38%) as severe, and only 35 (21.87%) had mild autism. CARS-2 ST T-scores were compared for severity based on the rating provided in the manual. There were 16 (10%), 19 (12%), 40 (25%), 37 (23%), and 48 (30%), counted as having very low, low, average, high, or very high severity of autism symptoms, respectively, with a median CARS-2 ST T-score of 55 with IQR of 16. The T-score-based grading did not have any values for extreme or minimal-no symptoms grading.

CARS-2 ST grading based on the raw scores was heavily skewed toward a "severe" rating resulting in more numbers toward the severe grading. However, the frequencies of ISAA grading were more equitable in the three groups with adequate numbers in all the subgroups. Thus the two clinical rating scales showed a difference when the same persons evaluated clinically the same child with the two tools. Further, CARS-2 ST raw scores overlapped categorical variables clubbed as no-mild symptoms, mild-moderate, and severe; hence, it is confusing to analyze it with values of synucleins, their fold changes, and ratios. Therefore the CARS-2 ST T-score was also analyzed.

Synuclein levels in autism

Since reference values were not available for the serum levels of α- and β-synuclein in children, a control group was selected, and ELISA was done to find values in normal children. α-synuclein control values were 29.47 ± 18.62 ng/ml (range: 22.39- 36.56). β-synuclein control values were 1219.16 ± 177.10 ng/ml (range: 1151.79-1200.52). The difference of both the α-synuclein and β-synuclein between cases and control was significant with a p < 0.001.

The mean α-synuclein in nano-gram/milliliters (ng/ml) was 5.02 ± 0.586 (range: 3.13-6.0 ng/ml); the median value 5.12 (IQR: 4.712-5.528) was considered for further analysis as it failed normality test. The mean β synuclein was 1424 ng/ml ± 122 (range: 1229-1616 ng/ml), and its median values were as seen in Table 2. Since the unit input into the Biorad ELISA machine for values of α-synuclein was to be measured in pg/ml and the β-synuclein was to be measured in ng/ml, the α-synuclein (pg/ml) was converted to ng/ml. To avoid the inherent deficits that may creep in when micro amounts are converted from one unit to another, it was decided to consider the fold changes in addition to the absolute values of α and β-synucleins.

**Table 2:** Descriptive parameters of synucleins Since the data did not follow normal distribution, median with IQR was considered for analysis of rest of the study. IQR: interquartile range

Parameters	α-synuclein	α-fold decrease	β-synuclein	β-fold increase	β/α
N	160	160	160	160	160
Mean (ng/ml)	5.02	5.88	1424	1.16	246
Median (ng/ml)	5.12	5.66	1415	1.16	246
Standard deviation	0.586	0.889	122	0.0994	35.9
IQR (25th-75th centile)	4.17-5.53	5.19-6.13	1169-1661	0.992-1.33	198-294
Minimum	3.13	4.83	1229	1.01	143
Maximum	6.00	9.27	1616	1.33	316

Synucleins and autism severity

Comparison of median values (interquartile range (IQR)) of the different synuclein with different grades of autism severity by ISAA and CARS-2 ST severity grading were tabulated as in Table 3.

**Table 3:** Median (IQR) values of the synucleins versus grades of autism severity by ISAA and CARS-2 ST The interquartile range is calculated as 25th percentile - (1.5*IQR) for the lower boundary and 75th percentile + (1.5*IQR) for the upper boundary. IQR: interquartile range; CARS: Childhood Autism Rating Scale-2 Standard Version; ISAA: Indian Scale for Autism Assessment

Descriptives	ISAA grade	β-synuclein	α-synuclein	β-fold increase	α-fold decrease	β/α
Number (N)	1	35	35	35	35	35
	2	70	70	70	70	70
	3	55	55	55	55	55
Median (ng/ml)	1	1418	5.68	1.16	5.11	272
	2	1396	5.13	1.15	5.65	246
	3	1425	4.79	1.16	6.06	238
IQR	1	109	0.589	0.0898	0.561	27.5
	2	257	0.220	0.174	0.243	36.0
	3	246	0.740	0.203	0.981	41.0
Minimum (ng/ml)	1	1268	4.85	1.04	4.83	212
	2	1231	4.79	1.01	5.37	214
	3	1229	3.13	1.01	5.55	143
Maximum (ng/ml)	1	1603	6.00	1.31	5.98	316
	2	1616	5.40	1.33	6.06	296
	3	1600	5.23	1.31	9.27	288
Descriptives	CARS-2 ST grade	β-synuclein	α-synuclein	β-fold increase	α-fold decrease	β/α
Number (N)	1	16	16	16	16	16
	2	26	26	26	26	26
	3	118	118	118	118	118
Median (ng/ml)	1	1381	5.82	1.13	4.98	285
	2	1448	5.24	1.19	5.54	261
	3	1412	5.02	1.15	5.78	239
IQR	1	101	0.0890	0.0828	0.0752	16.0
	2	82.1	0.254	0.0672	0.269	16.8
	3	252	0.404	0.205	0.472	39.0
Minimum (ng/ml)	1	1361	5.81	1.12	4.83	272
	2	1268	4.85	1.04	5.11	212
	3	1229	3.13	1.01	5.37	143
Maximum (ng/ml)	1	1582	6.00	1.30	4.99	316
	2	1603	5.68	1.31	5.98	294
	3	1616	5.40	1.33	9.27	296

**Figure 1:**
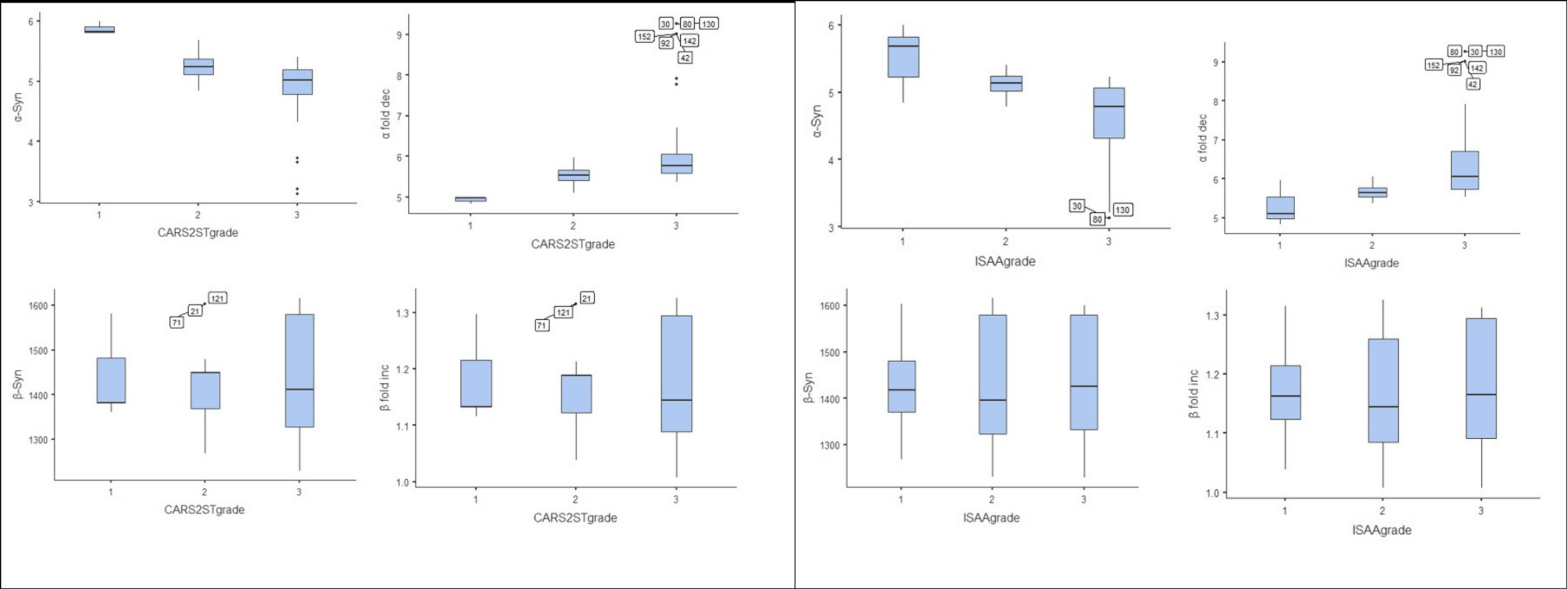
Boxplot analysis of ISAA and CARS-2 ST grades with synucleins and their fold changes Median (horizontal line inside box), 25th and 75th centiles (lower and upper margin of the box), and outliers (dots or values outside the box). ISAA: Indian Scale of Autism Assessment; CARS-2 ST: childhood autism rating scale-2 standard test

Since the data did not follow parametric distribution, a box plot was used to compare distributions of severity grading variable and α-synuclein levels or its fold changes, and outliers were identified as in Figure 1.

Correlation analysis of synuclein levels with ISAA and CARS-2 ST grades

Correlation analysis was done to evaluate the strength and direction of the relationship between synucleins and the grades of severity. Correlation analysis of the severity rating by raw score between CARS-2 ST or ISAA and α-synuclein, β-synuclein, β-fold increase, α-fold decrease, and β/α ratio, showed that β-synuclein or its fold increase did not show any significant Spearman’s correlation between any of the above parameters except β/α. However, α-synuclein and its fold decrease had a significant negative correlation with CARS-2 ST raw score, and ISAA, but a positive correlation with β:α (Table 4).

**Table 4:** Correlation matrix of synuclein levels with CARS-2 ST and ISAA Note: ^*^p < 0.05, ^**^p < 0.01, ^***^p < 0.001. Since the data did not follow normal distribution, Spearman's coefficient was calculated. ISAA: Indian Scale of Autism Assessment; CARS-2 ST: childhood autism rating scale-2 standard test

Molecular markers		β-Syn	α-Syn	β-fold inc	α-fold dec	β/α	CARS-2 ST grade	ISAA grade	CARS-2 ST T-score
β-Syn	Spearman's rho	—	—	—	—	—	—	—	—
	df								
	p-value								
α-Syn	Spearman's rho	0.067	—	—	—	—	—	—	—
	df	158							
	p-value	0.398							
β-fold increase	Spearman's rho	0.980^***^	0.110	—	—	—	—	—	—
	df	158	158						
	p-value		0.167						
α-fold decrease	Spearman's rho	-0.067	-1.000^***^	-0.110	—	—	—	—	—
	df	158	158	158					
	p-value	0.398		0.167					
β/α	Spearman's rho	0.702^***^	-0.694^***^	0.717^***^	-0.694^***^	—	—	—	—
	df	158	158	158	158				
	p-value								
CARS-2 ST grade	Spearman's rho	-0.059	-0.562^***^	-0.086	0.562^***^	-0.460^***^	—	—	—
	df	158	158	158	158	158			
	p-value	0.460		0.278					
ISAA grade	Spearman's rho	-0.030	-0.693^***^	-0.068	0.693^***^	-0.521^***^	0.750^***^	—	—
	df	158	158	158	158	158	158		
	p-value	0.706		0.394					
CARS-2 ST-T-score	Spearman’s rho	-0.172^*^	-0.778^***^	-0.207^**^	0.778^***^	-0.654^***^	0.772^***^	0.891^***^	—
	df	158	158	158	158	158	158	158	
	p-value	0.030	<0.001	0.009	<0.001	<0.001	<0.001	<0.001	

One-way ANOVA (Kruskal-Wallis test) analysis was done, since the data were nonparametric, to find whether the synucleins or their fold changes change with grades of severity of autism (ISAA grades). The effect size was mild (<0.3) in the parameters. Using the Kruskal Wallis test there was a significant difference in α-Syn, α-fold decrease, and β/α ratio across the three grades of ISAA with χ^2^ = 76.3, 76.3, and 43.76, respectively, p < 0.001 each, whereas the p-value for β-synuclein and β-fold increase was 0.647 and 0.541, respectively. Thus, at least two groups in the above were different significantly. Since there were more than five groups in the study, determining which of these groups differed from each other was done using a post hoc test. Dwass-Steel-Critchlow-Fligner (DSCF) pairwise comparison was done as a post hoc test; α-synuclein, α-fold decrease, and β/α ratio had a p-value of <0.001 in all pairs and for β-synuclein and β-fold increase, p-value returned as not significant in all pairs (p = 0.46, 0.95, 0.96).

Similar analysis with CARS-2 ST severity grading done using the Kruskal Wallis test showed a significant difference in α-synuclein, its fold decrease, and β/α ratio across the three CARS-2 ST grades of severity with χ^2^(2) = 56, 56, 38.82, respectively, p < 0.001, ϵ2 = 0.35, 0.35, and 0.24, respectively, and then same was confirmed by DSCF pairwise post-hoc testing. Effect size is mild in the parameters. The p-value for β-synuclein and β-fold increase was not significant (p = 0.753; 0.541). DSCF pairwise comparison also did not return any significant difference.

Synucleins as a possible marker of autism

Receiver operating characteristics (ROC) are used to quantify how accurately synucleins can discriminate between children with autism and non-autistic children. The cut-off value was obtained to identify the disorder along with sensitivity, specificity, PPV, NPV, and area under the curve (AUC). The results are tabulated below in Table 5.

**Table 5:** ISAA values and synuclein markers Though AUC returned significance with β-synuclein also, post-hoc analysis showed no significant difference with pair-wise comparison. PPV: positive predictive marker; NPV: negative predictive value; AUC: area under the curve

Marker	Cut-off value	Sensitivity	Specificity	PPV	NPV	AUC	DeLong-post-hoc p-value
α-synuclein	5.318	93.6%	74.3%	92.6%	76.5%	0.868 (0.792-0.94)	<0.001
α-fold decrease	5.453	93.6%	74.3%	92.6%	76.5%	0.868 (0.792-0.94)	<0.001
β-synuclein	1339.42	38.4%	91.4%	94.1%	29.4%	0.546 (0.452-0.64)	0.167
β-fold increase	1.0982	40.8%	91.4%	94.4%	30.2%	0.561 (0.468-0.655)	0.099

**Figure 2:**
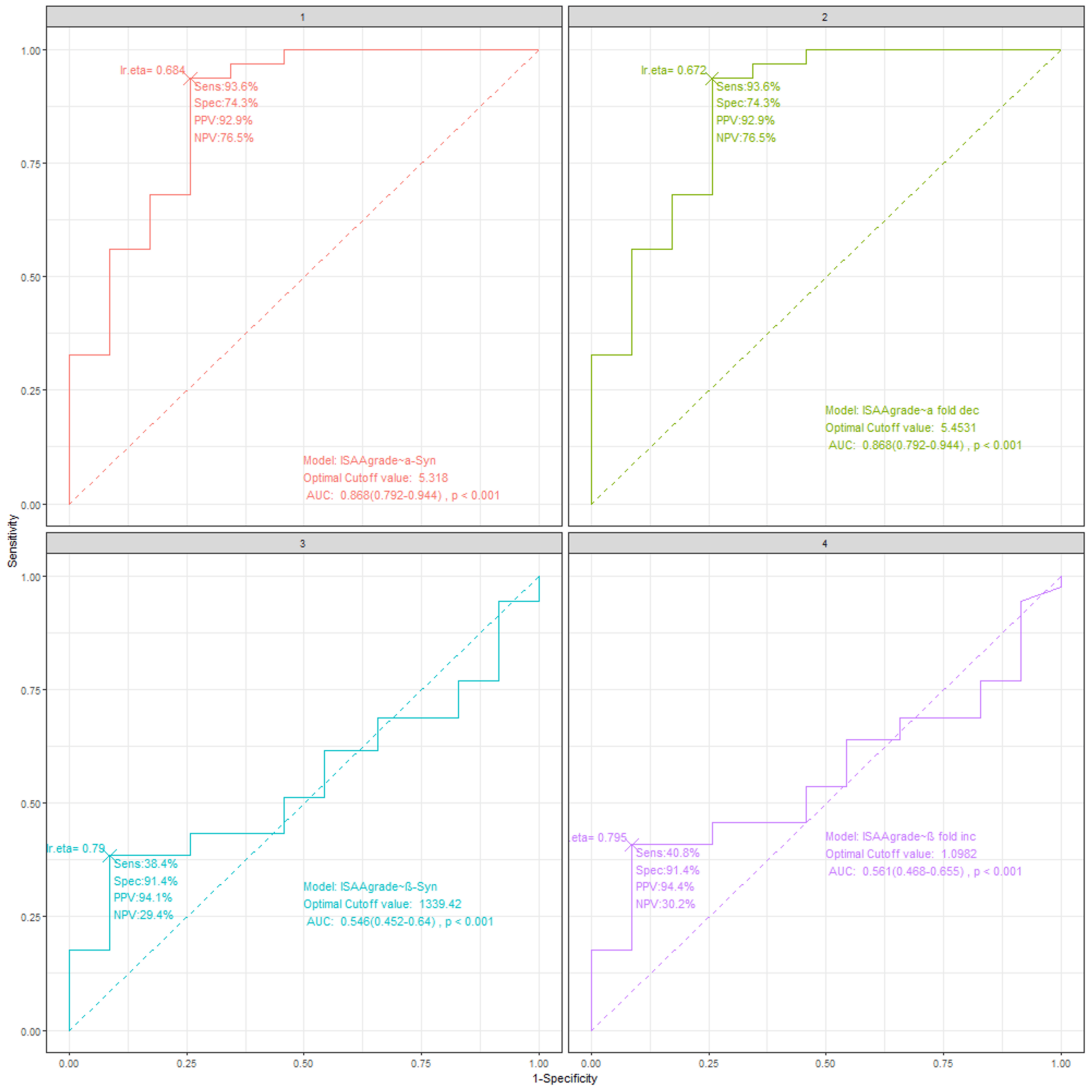
ROC analysis of synucleins for autism diagnosis with ISAA For α synuclein and α synuclein fold decrease, sensitivity was 93.6%, specificity was 74.3%, PPV was 92.9%, and NPV was 76.5% with a cutoff value of 5.38 and 5.45, respectively. ROC: receiver operating characteristics; AUC: area under the curve; ISAA: Indian Scale for Autism Assessment; PPV: positive predictive value; NPV: negative predictive value

When ISAA grading of severity was compared with different markers, only α synuclein and its fold decrease were found to be significantly different in ROC analysis and its post hoc test (DeLong test) (Figure 2).

Compared to ISAA, the CARS-2 ST grading showed a different picture with α-synuclein, as shown in Table 6 below. A cut-off of 5.68 ng/ml α-synuclein could identify the mildest case of autism identified by CARS-2 ST. Similarly, at 5.1056 ng/ml α-fold decrease also could identify all the cases graded by CARS-2 ST (p < 0.001). Post-hoc testing of groups also was significant (p < 0.001) (Table 6).

**Table 6:** ROC analysis - CARS-2 ST and cut-off of α-synuclein, β-synuclein, and their fold changes At a cut-off of 5.68 ng/ml for α-synuclein, all cases of autism diagnosed by CARS-2 ST were covered. The fold decrease of α-synuclein at a cutoff of 5.1056 ng/ml picked up all autistic children, even the mildest grade of autism, without compromising on specificity. PPV: positive predictive value; NPV: negative predictive value; AUC: area under the curve; CARS-2 ST: childhood autism rating scale-2 standard test; ROC: receiver operating characteristics

Markers vs. CARS-2 ST	Cut-off value	Sensitivity	Specificity	PPV	NPV	AUC	DeLong-post-hoc p-value
α-synuclein	5.68	100%	100%	100%	100%	1 (1-1)	<0.001
α-fold decrease	5.1056	100%	100%	100%	100	1 (1-1)	<0.001
β-synuclein	1350.81	37.5%	100%	100%	15.1%	0.531 (0.362-0.0.576)	0.284
β-fold increase	1.013	40.8%	91.4%	94.4%	30.2%	0.544 (0.352-0.559)	0.201

**Figure 3:**
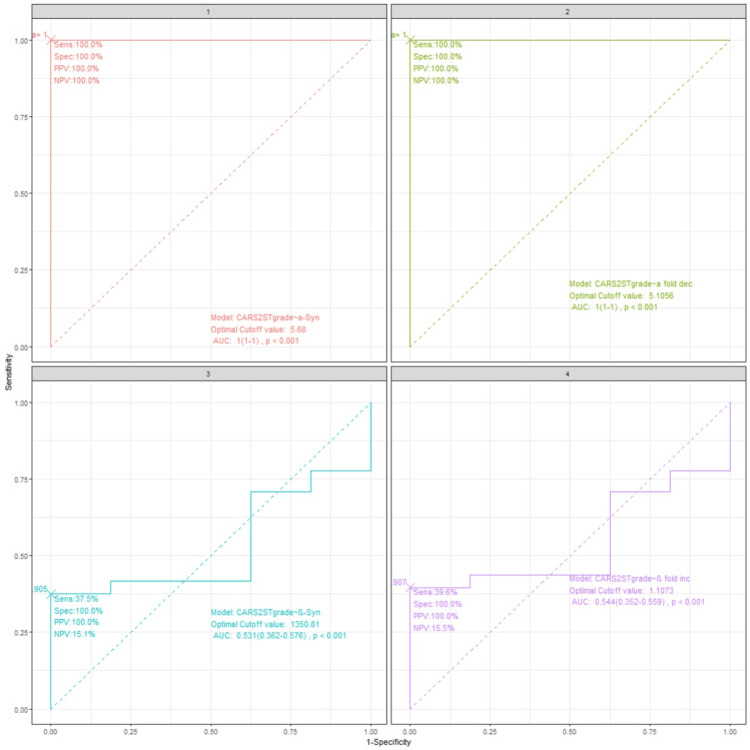
ROC analysis - CARS-2 ST and synucleins PPV: positive predictive value; NPV: negative predictive value; AUC: area under the curve; CARS-2 ST: childhood autism rating scale-2 standard test; ROC: receiver operating characteristics

Though the p-value for AUC of β-synuclein and β-synuclein fold increase was <0.001 in the ROC analysis, post-hoc analysis using the De-Long test showed no significance for both. Hence the medians of all the groups in CARS-2 ST are not different from each other, so there was no need to find out which groups are different from each other. Hence β-synuclein may not be ideal for comparing with different grades of CARS scoring of severity (Figure 3).

## Discussion

The present study was conducted to find the relation between synucleins and to assess the possibility of using them as a biomarker. Since the children were sex-matched, an almost equal number of children of both sexes were enrolled in the study, and the significance of the sex on the values was not assessed. However, previous studies have already established the possibility of sex influencing the values of α-synuclein [3]. Contrary to the clinical criteria required to establish the diagnosis of autism, where stereotypic behaviors are mandatory, parents hardly reported it as a presenting complaint during the interview. Their primary concern was a lack of preverbal skills like poor eye contact, lack of name-call response (40%), and poor speech (32%). There was no significant difference in the birth order and ASD symptoms in the present study. This is in contrast to many other studies that found that earlier birth order was associated with a higher incidence of ASD [16,17]. There was a disparity between the two rating scales in rating the severity - CARS-2 ST raw score severity and ISAA severity - with 73.8% reported as severe when the CARS-2 ST raw score rating was used and only 34.38% as severe in ISAA. This probably was due to the clubbing of categorical variables in classification criteria, where CARS-2 ST used no-, mild, mild-moderate, and severe as classification, and ISAA used mild, moderate, and severe as separate entries. Hence, the T-score severity rating of CARS-2 ST was used, which compared the symptoms the index child had, with other children having ASD.

Synuclein Values as a Potential Marker of Autism

Compared to normal children, children with autism had very low levels of α-synuclein (mean: 29.47 ± 18.62 ng/ml vs. 5.02 ± 0.586 ng/ml; p < 0.001). However opposite results were obtained with β synuclein (cases: 1219.16 ± 177.10 ng/ml; control: 1424 ng/ml ± 122 ng/ml). This shows that synuclein, especially α-synuclein is affected in children with autism and can be a potential marker of the presence of disease. It is a known fact that α-synuclein is deranged in many neurodegenerative disorders including adult disorders like Parkinson's disease in which it was found to be elevated. However, in autism, it was consistently found to be reduced when compared with normal children. The utility of these findings as a marker requires a detailed analysis of their predictive values, sensitivities, and specificity.

Synucleins and Severity of Autism

α-synuclein values decreased as the severity levels increased in ISAA and CARS-2 ST ratings (Table 3). This is similar to a previous study [18], though other studies disagree with this proposition [3]. Such a contradiction wherein the values were reported as increased may be due to the sample selection process in the previous study, where they did not consider other factors that may alter synuclein levels in the blood. Utmost care was taken to exclude the maximum confounding clinical conditions, including the possibility of late effects of COVID-19 in the brains of these children, which had affected our sample size, even though the study duration was long enough. However, like in the previous study, though an increasing trend of β-synuclein was obtained in the present study, a clear-cut statistically significant relationship could not be arrived at with it. The trend of decreasing α-synuclein values was secular in the age group from 2-8 years in the present study. This was similar to many earlier studies, including the one by Sriwimol and Limprasert in 2018 [18]. They concluded that β-synuclein levels were significantly different (p < 0.05) between control and ASD patients. α-synuclein was decreased proportionately to ASD severity; β-synuclein was found to be increased, though not significantly correlate with the severity of ASD. β-synuclein is a protein predominantly found in the neocortex, hippocampus, striatum thalamus, and cerebellum while α-synuclein is found in the CNS and red blood cells. Hence, it may be possible that β-synuclein may be raised in ASD as it functions as a neuroprotector against α-synuclein-induced neurotoxicity and prevents the aggregation of α-synuclein. The aggregation of α-synuclein in the neurons in the CNS may also lead to decreased levels in the blood [19]. There are recent reports that support the lower α-synuclein levels in ASD children. It was found that these children had decreased expression of the synuclein-α gene (SCNA gene), leading to lower production of its protein, viz., α-synuclein [20]. Further, 99% of α-synuclein in peripheral blood is found in RBCs. Hence, the changes reflected in the study population's plasma reflect the CNS changes [21].

Correlation Between Synucleins and CARS Grading of Severity

A correlation matrix using Spearman’s coefficient was used to evaluate the strength and direction of the relationship between synucleins and grades of autism by CARS-2 ST and ISAA without the requirement of a consistent increase with each grade. When the correlation of the synuclein fold changes with the severity of autism was assessed, the α-synuclein fold changes had a significant inverse correlation with increasing severity of autism in CARS-2 ST (rho: -0.562), CATS-2ST T-score (rho: -0.778) and ISAA (rho: -0.693). β/α showed a moderately strong correlation when compared with α-Synuclein and its fold decrease. However, β-synuclein did not demonstrate any direct correlation. To our knowledge, no studies have considered the fold changes, which could avoid any discrepancies that may creep up during unit conversions. Also, the absolute values may vary depending on the ELISA kit used; hence, fold changes rather than absolute values may be a better way to compare the correlation with severity. Further, once the diagnosis is made as ASD, the severity of autism symptoms should be compared with those who have similar symptoms. This will help to objectively assess the improvement following interventions rather than the current method of using subjective raw score-based scoring systems. Except with β synuclein and its fold increase, CARS-2 ST T-score correlated with all parameters. A β/α ratio also showed a significant correlation (p < 0.001) to CARS-2 ST severity grading, T-score, β-fold increase, and α-fold decrease. So, α-synuclein, its fold decrease shows a significantly strong negative correlation with all the grades of severity measured.

The area under the ROC curve (AUC) is a better measure for assessing the ability of a test to discriminate whether a specific condition is present or not. The overall diagnostic performance was evaluated with the ROC curve, which compared it with CARS-2 ST ratings. It showed that α-synuclein fold-decrease had a very good (0.93) discriminating value in ASD versus No ASD and its severity. Accordingly, the cut-off value was 5.68, which was 85.7% sensitive and 86.7% specific with a PPV of 93.8%. β-synuclein fold-increase was found to have no such discriminating value with an AUC of 0.54. So far, no such studies are available assessing the AUC of the fold changes of these synucleins for the diagnosis or severity rating of ASD.

Strengths of study

This study indicates that α-synuclein may be used along with clinical tools like CARS-2 ST or ISAA as an objective measure in the diagnosis of ASD. Though synucleins are altered in many neurodegenerative disorders, the peculiar aspect of a decrease in α-synuclein and simultaneous increase of β-synuclein may be used to add strength to the clinical diagnosis.

Further, the present study brings out the fact that α-synuclein and its fold changes decrease in children with autism whereas β-synuclein increases. The fact that α-synuclein correlated with the severity of CARS-2 ST T-score and ISAA severity rating shows that it can also be used as a marker for documenting the progress in clinically diagnosed autism and its severity. However, a study with a larger sample size done in a multi-centric way on a multi-ethnic population across age, sex, and other factors will increase its reliability to be used as an objective tool for severity rating and measuring the improvement following interventions. This may lead to the manufacturing of point-of-care devices using the synucleins, leading to better objective measurements. This study provides a basis for such considerations also.

Limitations of the study

This study was done on age and sex-matched children of 2-8 years old. Autism is a neurodevelopmental disorder with strong male preponderance (4:1; male: female). Hence, the effect of these parameters, especially sex, on synuclein values or its fold changes has to be pondered further with larger studies with matching population ratios. Symptoms of autism are known to change with age; hence, its effect on synucleins also needs to be studied on a larger population to make this simple method of objective assessment for routine clinical use in assessing the severity of age. As the symptoms change with interventions, future studies may be undertaken to find the effect of interventions on synucleins, so that a biomarker may be made available for objectively assessing the improvement in symptoms. The values of α-synuclein were initially measured in pg/ml which was later converted to ng/ml as the β-synuclein levels were expressed in ng/ml. This was done to ensure uniformity in values. Care was taken to avoid the effect of unit conversions, especially on microvalues. However, fold changes were also calculated for both synucleins and compared with severity rating scales with which the values of synucleins were also analyzed. This ensured that the unit conversions did not affect the values compared.

## Conclusions

Absolute values of α-synuclein in the blood correlated with ASD severity and had a good discriminatory value when compared with β-synuclein. However, the fold decrease in α-synuclein had better utility as it avoided many biases and confounders due to factors connected with it and discrepancies associated with their respective unit conversions. A β/α ratio of the values also has similar discriminatory effects in assessing the severity of ASD. Fold changes may give a better objective assessment of improvement following interventions as they correlated well with the severity of symptoms of similar autistic children, as evidenced by a good AUC with the T-score of CARS-2 ST. However, a study with a larger age-wise distribution may assess its significance over time, as ASD symptomatology changes with age, even in the same patient. Further studies are needed to understand the effect of sex on these values, especially since ASD has a 4:1 male-to-female ratio. Further, it has to be understood that α-synuclein, though predominantly present in the brain, can also be present in RBCs. This has been considered in the present study by using the plasma, taking values from normal age-matched children, and using them as the control values.

It is prudent to use the T-score-based severity rating of CARS-2, especially while assessing the changes in autism symptomatology over time, rather than the severity rating based on the raw score, as is widely practiced by clinicians. Including α-synuclein fold change with a T-score rating of CARS-2 gives better, objective, evidence-based criteria to assess the severity while assessing the effect of interventions. Diagnosing mild ASD, which is often missed/doubtful, may benefit from simultaneous measurement of α-synuclein.

## References

[1] Okoye C, Obialo-Ibeawuchi CM, Obajeun OA (2023). Early diagnosis of autism spectrum disorder: a review and analysis of the risks and benefits. Cureus.

[2] Raghavan K, Dedeepiya VD, Ikewaki N, Sonoda T, Iwasaki M, Preethy S, Abraham SJ (2022). Improvement of behavioural pattern and alpha-synuclein levels in autism spectrum disorder after consumption of a beta-glucan food supplement in a randomised, parallel-group pilot clinical study. BMJ Neurol Open.

[3] Al-Mazidi S, Al-Ayadhi LY (2021). Plasma levels of alpha and gamma synucleins in autism spectrum disorder: an indicator of severity. Med Princ Pract.

[4] Cheng F, Vivacqua G, Yu S (2011). The role of α-synuclein in neurotransmission and synaptic plasticity. J Chem Neuroanat.

[5] Barba L, Paolini Paoletti F, Bellomo G (2022). The alpha and beta synucleins: from pathophysiology to clinical application as biomarkers. Mov Disord.

[6] El-Ansary A, Alhakbany M, Aldbass A, Qasem H, Al-Mazidi S, Bhat RS, Al-Ayadhi L (2021). Alpha-Synuclein, cyclooxygenase-2 and prostaglandins-EP2 receptors as neuroinflammatory biomarkers of autism spectrum disorders: Use of combined ROC curves to increase their diagnostic values. Lipids Health Dis.

[7] Huang M, Wang B, Li X, Fu C, Wang C, Kang X (2019). α-synuclein: a multifunctional player in exocytosis, endocytosis, and vesicle recycling. Front Neurosci.

[8] Busch DJ, Oliphint PA, Walsh RB, Banks SM, Woods WS, George JM, Morgan JR (2014). Acute increase of α-synuclein inhibits synaptic vesicle recycling evoked during intense stimulation. Mol Biol Cell.

[9] Fan Y, Limprasert P, Murray IV (2006). Beta-synuclein modulates alpha-synuclein neurotoxicity by reducing alpha-synuclein protein expression. Hum Mol Genet.

[10] Surguchev AA, Surguchov A (2017). Synucleins and gene expression: ramblers in a crowd or cops regulating traffic?. Front Mol Neurosci.

[11] George JM (2002). The synucleins. Genome Biol.

[12] Chattopadhyay N (2024). Autism screening in India: many a chasm to bridge. Indian Pediatr.

[13] Lefort-Besnard J, Vogeley K, Schilbach L, Varoquaux G, Thirion B, Dumas G, Bzdok D (2020). Patterns of autism symptoms: hidden structure in the ADOS and ADI-R instruments. Transl Psychiatry.

[14] Schopler E, Reichler RJ, DeVellis RF, Daly K (1980). Toward objective classification of childhood autism: childhood autism rating scale (CARS). J Autism Dev Disord.

[15] Chakraborty S, Thomas P, Bhatia T, Nimgaonkar VL, Deshpande SN (2015). Assessment of severity of autism using the Indian scale for assessment of autism. Indian J Psychol Med.

[16] Andoy Galvan JA, Ramalingam PN, Patil SS, Bin Shobri MA, Chinna K, Sahrir MS, Chidambaram K (2020). Mode of delivery, order of birth, parental age gap and autism spectrum disorder among Malaysian children: a case-control study. Heliyon.

[17] Banerjee N, Adak P (2022). Birth related parameters are important contributors in autism spectrum disorders. Sci Rep.

[18] Sriwimol W, Limprasert P (2018). Significant changes in plasma alpha-synuclein and beta-synuclein levels in male children with autism spectrum disorder. Biomed Res Int.

[19] Hayashi J, Carver JA (2022). β-Synuclein: an enigmatic protein with diverse functionality. Biomolecules.

[20] Karaca M, Tahtasakal R, Dana H (2023). Decreased levels of alpha synuclein in families with autism spectrum disorder and relationship between the disease severity. Brain Res.

[21] Barbour R, Kling K, Anderson JP (2008). Red blood cells are the major source of alpha-synuclein in blood. Neurodegener Dis.

